# Treatment of Alzheimer's Disease with Anti-Homocysteic Acid Antibody in 3xTg-AD Male Mice

**DOI:** 10.1371/journal.pone.0008593

**Published:** 2010-01-20

**Authors:** Tohru Hasegawa, Nobuyuki Mikoda, Masashi Kitazawa, Frank M. LaFerla

**Affiliations:** 1 Saga Women's Junior College, Saga City, Saga, Japan; 2 Kyudo Ltd., Tosu City, Saga, Japan; 3 Department of Neurobiology & Behavior, University of California Irvine, Irvine, California, United States of America; University of North Dakota, United States of America

## Abstract

Alzheimer's disease (AD) is an age-associated progressive neurodegenerative disorder with dementia, the exact pathogenic mechanisms of which remain unknown. We previously reported that homocysteic acid (HA) may be one of the pathological biomarkers in the brain with AD and that the increased levels of HA may induce the accumulation of intraneuronal amyloid-beta (Aβ) peptides. In this study, we further investigated the pathological role of HA in a mouse model of AD. Four-month-old prepathological 3xTg-AD mice exhibited higher levels of HA in the hippocampus than did age-matched nontransgenic mice, suggesting that HA accumulation may precede both Aβ and tau pathologies. We then fed 3-month-old 3xTg-AD mice with vitamin B6-deficient food for 3 weeks to increase the HA levels in the brain. Concomitantly, mice received either saline or anti-HA antibody intraventricularly via a guide cannula every 3 days during the course of the B6-deficient diet. We found that mice that received anti-HA antibody significantly resisted cognitive impairment induced by vitamin B6 deficiency and that AD-related pathological changes in their brains was attenuated compared with the saline-injected control group. A similar neuroprotective effect was observed in 12-month-old 3xTg-AD mice that received anti-HA antibody injections while receiving the regular diet. We conclude that increased brain HA triggers memory impairment and that this condition deteriorates with amyloid and leads to subsequent neurodegeneration in mouse models of AD.

## Introduction

Amyloid plaques and neurofibrillary tangles are the two pathophysiological hallmarks of Alzheimer's disease (AD). Intracellular amyloid-beta 42 (Aβ42) is increasingly being recognized as an early pathological trigger that can lead to amyloid plaques and may even induce neurofibrillary tangles. We previously reported that homocysteic acid (HA) induces intracellular accumulation of Aβ42 and that the production of α-synuclein in the presence of methionine results in cell death [1], [19].

HA affects the two pathophysiological hallmarks of AD and may be involved in its etiology. HA is also known as an NMDA (N-methyl-D-glutamate) receptor agonist [2] and is released under mental stress from astrocytes by the activation of β-adrenergic receptors [3]. Also, previous studies have reported HA-induced neurodegeneration by oxidative stress [4] and mitochondrial respiration inhibition [5].

On the basis of these HA toxicities, few researchers have studied the possibility of HA pathogenicity in the onset of AD [6]. In humans, it is reported that urinary excretion of homocysteine is much slower than that of mice [7], which suggests that humans may produce greater HA level than mice, since HA is known to be produced from homocysteine.

The 3xTg-AD mouse model showed memory impairment at an early age because of Aβ accumulation in neuronal cells [8], [9]. In this study, we studied 3xTg-AD mice to further investigate the role of HA in the development of AD-related pathologies in the brain. We observed an increased level of HA in the hippocampus of 4-month-old 3xTg-AD homozygous mice compared with the level of age-matched control mice. The brain HA production in the 3xTg-AD mice was increased by feeding them with a vitamin B6-deficient diet for 3 weeks. These pathological changes as well as cognitive impairments were significantly mitigated by concomitant intracranial injections of anti-HA antibody in these mice. Our findings suggest that increased HA may partly contribute to the progression of AD in the mouse model.

## Materials and Methods

### 3xTg-AD mice

The mouse germline used in this study was a kind gift from Professor F. M. Laferla (University of California, Irvine). The housing environment (12 h/12 h light/dark cycle) was a germ-free clean room. Seven 3xTg-AD hemizygous male mice (3 and 7 months old) were studied. Also, four nontransgenic (non-Tg) mice were studied. The 3xTg-AD mice developed both plaque and tangle pathology in AD-relevant brain regions. Despite an equivalent overexpression of the human sAPP and human tau transgenes, the 3xTg-AD mice developed extracellular As deposits before tangle formation, consistent with the amyloid cascade hypothesis. In addition, these mice exhibited deficits in synaptic plasticity, including long-term potentiation, which occurs before extracellular As deposition and tau pathology but is associated with intracellular As immunoreactivity. These results support the view that synaptic dysfunction is a proximal defect in the pathobiology of AD and precede extracellular plaque formation and neurofibrillary pathology. As these 3xTg-AD mice phenocopy critical aspects of AD neuropathology, this model will be useful in preclinical intervention trials, particularly because the efficacy of anti-AD compounds in mitigating the neurodegenerative effects mediated by both signature lesions can be evaluated.

### Vitamin B6-Deficient Food

Vitamin B6-deficient food was purchased from Kyudo Ltd.

Nutrient composition will be described further.

### Anti-HA Antibody

Anti-HA antibodies were purchased from MoBiTec Co. (Germany). Polyclonal antisera were raised in rabbits after immunization with a glutaraldehyde-containing HA conjugate, following which antibody specificity was determined by performing ELISA with competition experiments involving HA-G-BSA, cysteine-G-BSA, and homocysteine-G-BSA (compound cross-reactivity ratios 1∶1, 1∶85, and 1∶231, respectively).

### HA Vaccine

We synthesized a glutaraldehyde-containing HA conjugate KLH (Keyhole limpet hemocyanin) compound, and mice were immunized twice by IP route with this compound and BCG(Bacillus Calmette-Guérin). Two weeks later KLH and BCG immunized mice again. Immunization volume was 20 µL.

### Morris Water Maze Test

The apparatus used for Morris water maze task comprised a circular aluminum tank (1.5 m in diameter) painted white and filled with water maintained at 26–29°C. The maze was located in a room containing several simple, visual extramaze cues. To reduce stress, mice were placed on a platform in both the hidden and cued versions of the task for 10 s before the first training trial.

### Spatial Reference Morris Water Maze Training

Mice were trained to swim to a 14-cm circular clear Plexiglas platform placed 1.5 cm below the water surface that was invisible to the mice while swimming. Platform location was randomly selected for each mouse but was kept constant for that mouse throughout the training period. In each trial, mouse was placed in the tank at one of the four designated starting points in a pseudorandom order. Mice were allowed to search for and escape to the submerged platform. If a mouse failed to find the platform within 60 s, it was manually guided towards it and allowed to remain there for 10 s. Then, each mouse was placed in a holding cage under a warming lamp for 25 s until the start of the next trial. To ensure that memory differences were not due to the lack of task learning, the mice underwent four trials a day for as many days as required to meet the criterion, which was defined as a <20-s mean escape latency before the first probe trial was run. To prevent overtraining, probe trials were run for each group as soon as they met the group criterion and stopped after all the groups met the criterion. Retention of spatial training was assessed 1.5 and 24 h after the last training trial. Both probe trials consisted of a 60-s free swim in the pool without the platform. Mice were monitored by a camera mounted on the ceiling directly above the pool and all trials were stored on videotape (burnt onto a DVD) for subsequent analysis. Parameters measured during the probe trial comprised initial latency time to reach the platform (1).

### Immunohistochemistry

Mice were sacrificed by CO_2_ asphyxiation, and the brains were rapidly removed and fixed for 48 h in 4% paraformaldehyde. Sections (50-µm thick) were processed for free-floating immunohistochemistry as previously described [8]. Anti-Aβ (6E10)-, anti-APP (22C11)-, and Aβ (40/42)-specific antibodies were applied overnight at 4°C. Sections were developed with diaminobenzidine (Vector Laboratories) substrate using the avidin–biotin–horseradish peroxidase system (Vector Laboratories). Quantification of Aβ was performed as described previously. Mice were excluded from the antibody group analysis (behavior and histology) if the cannulae were found to be placed incorrectly. To obtain the percentage difference between the antibody- and PBS-treated tissues (controls), we applied the following formula: number of pixels in the antibody-treated hippocampus − number of pixels in the PBS-treated hippocampus/number of pixels in the PBS-treated hippocampus. The number of pixels calculated in each case is the sum of five readings per mouse averaged across the entire group.

### HA Level Measurement

HA was extracted from mouse brain (hippocampus and cortex) with trichloroacetic acid. Brains (1.50−2.00 g) were isolated from 4-month-old 3xTg-AD homozygous male mice. Mice were killed by rapid decapitation and their brains were quickly excised and placed on an ice-cold petri dish. For the gradient high-performance liquid chromatography (HPLC) method, tissue samples were weighed and homogenized using a sonicator for 10 s in ice in 4 mL of ice-cold 10% (w/v) trichloroacetic acid per 100 mg tissue (wet weight). HA (4 µg) was added as an internal standard. For isocratic HPLC, tissue samples were divided into six aliquots. The samples were homogenized as described above. The homogenates for isocratic or gradient HPLC were left on ice for 1 h and centrifuged at 20,000×*g* for 25 min. The supernatant was washed five times with an equal volume of diethyl ether and the aqueous phase was maintained. Residual ether was evaporated under nitrogen at room temperature for 5 min. Immediately thereafter, 20 µL was injected into the HPLC system.

### Urinay HA Level

Urine of the 3xTg-AD male mice (15-month-old) was collected for 24 h. The urinary HA level was measured according to the method of the HPLC system.

### Ventricular Cannula

Mice were anesthetized as follows: 2-mm-wide incisions were made in the left hemisphere and a guide cannula was inserted into the left ventricular space using a Teflon tube (1 mm in diameter). This operation did not impair learning and memory performance, and the abilities of the operated mice were similar to those of mice that did not undergo surgery.

### Statistics

Statistical significance was estimated with Student's *t*-test and *P*-values (p<0.05).

All animals experiments have been done according to the accepted international guideline methods and Saga Woman Junior College approved this work according to animal experimental guideline.

## Results

### HA Levels in Mouse Model Brain

We measured HA levels in the brains of 4-month-old 3xTg-AD-homozygous mice. At this age, mice display an intracellular accumulation of Aβ in the brain regions affected by AD. This accumulation also appears to be associated with the early memory deficit exhibited by these mice [9]. As shown in Table 1, HA levels in the 3xTg-AD mice were significantly (*P*<0.05) higher than those in age-matched control mice. This finding indicates that HA may contribute to the pathology of AD.

**Table 1:** Homocysteic Acid Level in 3xTg-AD mice brain at 4 months compared with Non-Tg control mice.

Category	Results	P value
Hippocampus		
HA level pmoles/mg wet weight		
Control	23.63±9.2 (n = 5)	
3xTg-AD	41.25±5.4 (n = 4)	<0.001
Cortex		
Control	20.41±6.6 (n = 5)	
3xTg-AD	32.16±4.8 (n = 4)	<0.01

HA level in the brain of 3xTg-AD male mice at 4 months was measured by the method described in Materials and Methods. Control was non-Tg male mice at 4 months.

We then fed 3-month-old 3xTg-AD mice vitamin B6-deficient food for 3 weeks to increase HA levels in the brain. The brain HA levels were significantly increased following the 3-week vitamin B6-deficient diet (Table 2). Consistent with this result, the B6-deficient group exhibited memory impairment compared with the control group (the normal feeding group exhibited good memory performance; data not shown here).

**Table 2:** HA Level in 3xTg-AD mice brain after Ingesting Vitamin B6-Deficient food for 3 weeks.

Category	Results	P value
Control (n = 10)	42.0±8.1 pmoles/mg brain	
B6 deficient (n = 10)	65.2±15.0 pmoles/mg brain	<0.001
Anti-HA antibody Treatment (n = 10)	20.1±7.1 pmoles/mg brain	<0.001

Control mice: 3xTg-AD mice at 3 months and 3 weeks with normal feeding. Control mice showed good memory performance, but B6-deficient feeding group showed memory impairment. Whole brain was homogenized with physiological saline and HA level was measured, after the memory task, according to the method described in Materials and Methods.

To further examine the role of HA in the pathogenesis of AD, we administered anti-HA antibody intracranially through guide cannula during the course of the vitamin B6-deficient diet. Anti-HA antibody significantly decreased the HA level in 3xTg-AD mice (Table 2) and HA vaccine decreased the urinary HA level of 3xTg-AD mice at 15 months. (HA vaccine administered to 12-month-old 3xTg-AD mice, and three months later the urinary HA level was measured; Table 3) Also HA vaccine specifically decreased HA level in mice brain at 12 months old (Table 3b).

**Table 3:** HA level in 3xTg-AD mice.

A. HA Level in Urine of 3xTg-AD Mice at 15 Months (n = 5)		
Category	Results	P value
Control	22.5±8.5 µM	
Vaccine treatment	7.8±9.1 µM (35% of control)	<0.001
B. HA Level in 3xTg-AD Mice Brain at 12 Months		
Category	Results	P value
Control (n = 10)	71.5±20.3 pmoles/mg brain	
Vaccine treatment (n = 10)	30.6±18.4 pmoles/mg brain (43% of control)	<0.001

Note: A: Vaccine treatment was started at 12 months. Three months later, urine was collected an entire day and HA level was measured. 3xTg-AD male mice were all homozygous. HA level with vaccine treatment decreased with time, indicating that vaccine efficacy became stronger with time.

**Note: For reference, HA level in 12-month-old in 3xTg-AD male mice were observed.**

Note: B: After Morris water maze test, these mice were sacrificed and their brains were immediately freezed in liquid nitrogen. Next, HA level was measured with the method described in Materials and Method.

### Memory Experiment

**Figure 1:**
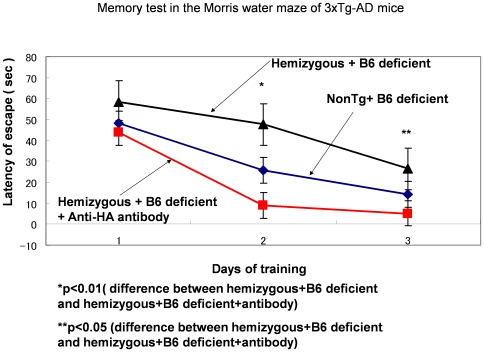
Long-Term Memory Test in the Morris Water Maze task. Non-transgenic mice had an average score of 3 mice each day. Hemizygous transgenic control (hemizygous + B6 deficient) mice had an average score of 3 transgenic control mice each day. Transgenic experimental (hemizygous + B6 deficient + anti-HA antibody) mice had an average score of 3 transgenic experimental mice each day. 3xTg-AD mice were aged 3 months and 3 weeks. Statistically significant difference was observed after 2 trial days between hemizygous + B6 deficient and hemizygous + B6 deficient + antibody (*P*<0.001) and after 3 trial days between hemizygous + B6 deficient and hemizygous + B6 deficient + antibody (*P*<0.05).

We next evaluated the hippocampus-dependent memory with the Morris water maze task. Three-month old 3xTg-AD mice given a vitamin B6-deficient diet exhibited significant memory impairment compared with 3-month-old control mice (non-Tg and Tg with a normal diet exhibited good memory performance; Fig. 1). Notably, cotreatment with anti-HA antibody significantly prevented vitamin B6-deficiency-induced memory impairment (Fig. 1). This result indicates that HA can induce memory impairment. Memory impairment induced by HA was greater in the presence of Aβ because transgenic control mice, in which there was Aβ-production and accumulation exhibited the worst memory impairment of the three groups (Fig. 1). Amyloid alone did not induce memory impairment despite the presence of higher levels of both Aβ-40 and -42 in the transgenic 3-month-old mice (hemizygous + B6 deficient + anti-HA antibody) compared with non-Tg mice [9]. Moreover, transgenic experimental mice exhibited better memory performance than non-Tg mice.

### Pathological Change

**Figure 2:**
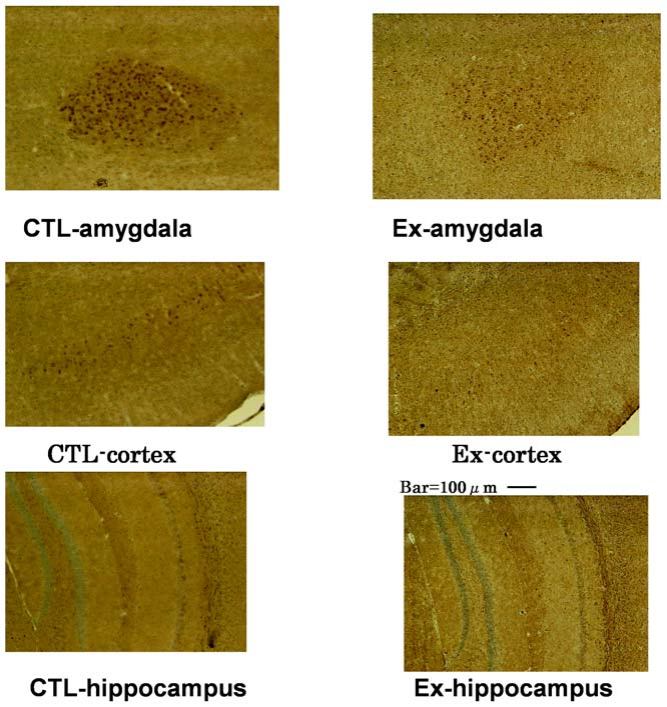
Immunohistochemical observations of Amygdalar, Cortical, and Hippocampal Neurons. Anti-HA antibody was diluted 100-fold. Immunohistochemical observations were repeated thrice and each observation gave the same result. Ten homozygous transgenic 3-month-old mice were fed B6-deficient food for 3 weeks. Transgenic experimental mice were injected with anti-HA antibody every 3 days. For details, see Materials and Methods. CTL, transgenic control mice; Ex, transgenic experimental mice.

Figure 2 demonstrates immunohistochemical staining for Aβ in the amygdalar, cortical, and hippocampal cells of transgenic control and transgenic experimental mice. Numerous stained neurons were seen in transgenic control mice, indicating Aβ accumulation in these neurons. In contrast, transgenic experimental mice (anti-HA antibody treatment) did not exhibit any stained neurons in the hippocampus and exhibited fewer stained neurons in the amygdala and cortex compared with transgenic control mice (those not treated with anti-HA antibody), indicating the inhibition of Aβ accumulation. Since we observed a preventive effect of anti-HA antibody in the acceleration of the pathogenesis of AD in 3xTg-AD mice induced by vitamin B6-deficient food (because 3-month-old 3xTg-AD mice with normal food intake did not exhibit any memory impairment, but 3-month-old 3xTg-AD mice with B6-deficient food exhibited strong memory impairment), we looked for a curative effect of anti-HA antibody on the established pathological changes in 3xTg-AD mice with AD. 3xTg-AD hemizygous 7-month-old male mice were fed vitamin B6-deficient food for 3 weeks (control) and anti-HA antibody (5 µL) was injected into their ventricular space every 3 days using guide cannula.

**Figure 3:**
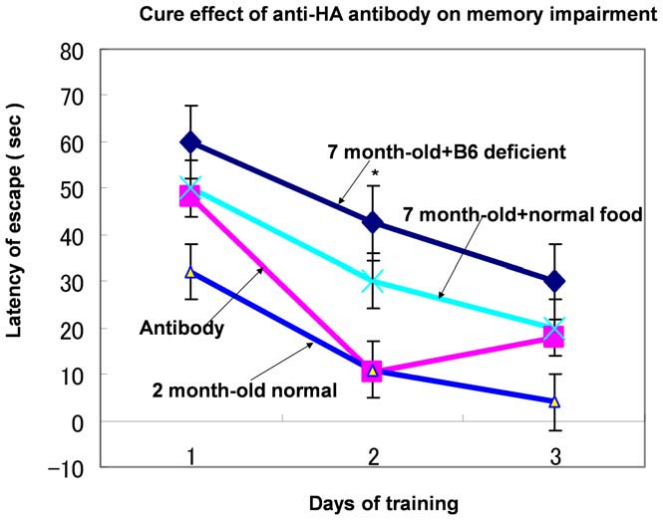
Curative Effect of Anti-HA Antibody as shown by Long-Term Memory Performance. Seven-month-old male 3xTg-AD homozygous mice mice fed B6-deficient food for 3 weeks served as the control. Experimental mice were treated with anti-HA antibody every 3 days; antibody (100-fold dilution) was injected into the brain as described in Materials and Methods. The figure shows average data for five male mice. To demonstrate the strong curative effect of the antibody, for comparison we show the results for 2-month-old hemizygous male mice, whose memory performance was normal. Statistically significant difference was observed after 2 trial days (*P*<0.001). However after 3 trial days, a statistically significant difference was not observed (^*^ *P*<0.001). The difference between control and experiment mice (*P*<0.01). The difference between experimental mice and 7-month-old mice with normal food (n = 5).

**Figure 4:**
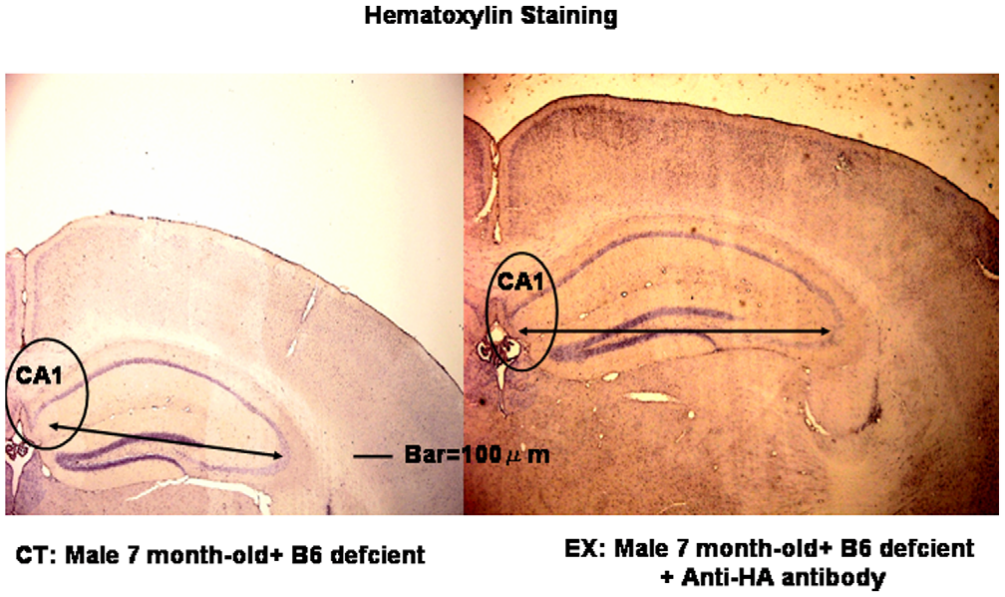
Hematoxylin-Eosin Staining of Cortical and Hippocampal Neurons in Control and Experimental Groups. Mice were treated the same as those shown in Fig. 3.

After 3 weeks, we measured memory performance in the Morris water maze tasks (Fig. 3), following which we evaluated the hematoxylin-eosin and immunohistochemical staining of the brain specimens. Control mice exhibited poor memory performance, but experimental mice injected with anti-HA antibody exhibited strong recovery of performance after 2 days of training, indicating good memory performance. Also, 7-month-old 3xTg-AD mice with normal food intake exhibited the same memory impairment; their memory impairment was not as severe as that of 3xTg-AD mice with vitamin B6-deficient food, which indicated that vitamin B6-deficient food accelerated memory impairment (data not shown here). Consistent with the results of the memory performance task, control mice accumulated Aβ in their neuronal cells in cortex, while experimental mice accumulated less Aβ than that of control mice. The result (data not shown) was the same as that shown in Fig. 2. Hippocampal volume was smaller in control mice compared with non-Tg hippocampal volume, but it recovered in the experimental group (Table 4; Fig. 4), which suggests that the control hippocampus showed neurodegeneration but the experimental mice may exhibit neurogenesis of hippocampus. It has recently been reported that the anti-pain effect induced neurogenesis of hippocampus [11]; on the basis of this report, we examined the anti-pain effect of HA vaccine on 21-month-old 3xTg-AD mice. Their tails were clipped (pain source) with a tiny metal clipper and they turned back to take off this clipper. The time they took to turn back to take off this clipper was measured. This result is shown in Table 5. Results of Table 5 clearly show that HA vaccine provides a strong anti-pain effect.

**Table 4:** The comparison of hippocampus major axis length between control and experiment.

Category	Results	Average	P value
Control	9, 8.8, 7.8, 9 8.2 (cm)	8.6±0.5	
Experiment	9.6, 10.1, 9.6, 10.3 (cm)	9.9±0.4	<0.01

Control: 7 month-old hemizygous + B6 deficient.

Experimental: 7 month-old hemizygous male +B6 deficient + anti-HA antibody.

5 different sections were observed and its major axis was measured.

Average (n = 2) is shown.

Non-Tg control showed Hippocampal major axis of 10±0.5 cm.

**Table 5:** Time taken to turn back and take off the clipper.

Category	Results (sec)	P value
Control	28.5±2 s (n = 5)	
Vaccine treatment	56±8 s (n = 5)	<0.001

Mice tails were clipped by a tiny metal clipper and the time taken by the mice to turn back and take off these clippers was measured. 3xTg-AD male mice were 18-month-old. Vaccine treatment was started at 12 months. 3xTg-AD male mice were all homozygous.

### Results (Normal Feeding for 3xTg-AD Mice)

**Figure 5:**
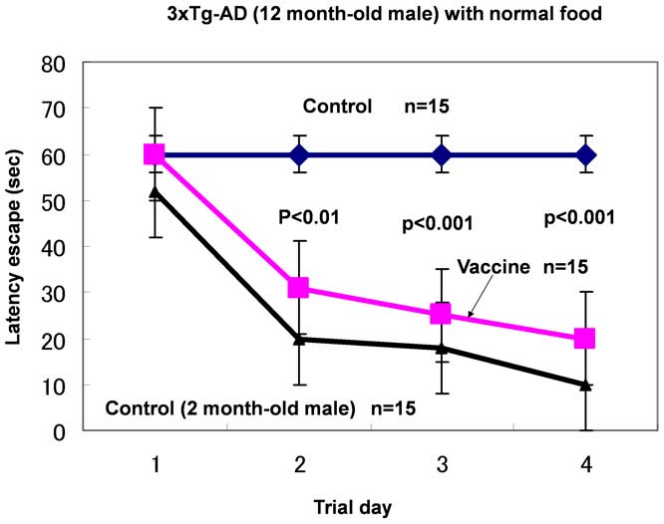
The Effect on Memory Performance of HA Vaccine on Normal Feeding of 3xTg-AD Male Mice. Mice were 12 months old. Memory performance was measured via the Morris water experiment described in Materials and Methods. Statistically significant difference was observed on the third, fourth, and fifth trial days (*P*<0.001). 3xTg-AD male mice were all homozygous (n = 15).

Our results were obtained by vitamin B6-deficient feeding to mice, which induced the vitamin B6-deficient burden on 3xTg-AD mice. We then investigated whether the cure effect of anti-HA antibody can be observed on 3xTg-AD mice fed with normal food. The result is shown in Fig. 5, and as clearly seen, there is a strong cure effect of HA vaccine in 3xTg-AD mice (12-month-old).

### The Effect of HA Vaccine Showed the Same Effect as of Anti-HA Antibody

We developed the HA vaccine according to the anti-HA antibody procedure described in Materials and methods. The effect of HA vaccine on memory impairment in 12-month-old 3xTg-AD mice is shown in Fig. 5. HA vaccine has a strong cure effect on memory impairment, which is the same as that of the anti-HA antibody.

## Discussion

We demonstrated the specificity of anti-HA antibody or HA vaccine to destroy HA. From Tables 2 and 3, it is clearly observed that anti-HA antibody and HA vaccine destroy HA in brain or urine, which indicates a decreased level of HA in brain and urine. B6-deficient food induces an increase in homocysteine, homocysteine sulfinic acid, and HA levels [10]. Recently, MacMahon et al. observed that lowering homocysteine with B vitamins did not improve cognitive performance [12] and that treating homocysteine levels should not be a focus of AD prevention. Homocysteine sulfinic acid is formed by the peroxidation of homocysteine [13], following which the latter does not play a role in the pathogenesis of AD, indicating that homocysteine sulfinic acid does not contribute to the disease pathogenesis of AD. Finally, because HA is formed from homocysteine and methionine [13], it may accelerate the pathogenesis of AD.

The results of our transgenic experiments confirmed that anti-HA antibody suppressed memory impairment induced by B6-deficient food. This hypothesis is supported by our data suggesting that higher levels of HA are present in 3xTg-AD homozygous mice compared with non-Tg mice (Table 1). Also, Table 2 shows the increased level of HA induced by vitamin B6-deficient feeding. The increased HA level was decreased by anti-HA antibody, which indicates that anti-HA antibody actually decreases the HA level. It is reported that HA itself has a neurodegenerative effect in immature rat [14]. Then why did the HA level increase in 3xTg-AD mice? We previously observed the production of HA by cystathionine-β-synthase (CBS) in the presence of calcium and peroxides such as lipid peroxides (unpublished observation). These observations suggest that 3xTg-AD mice exhibit the activated gene of presenilin, which induced the calcium level [18]. 3xTg-AD mice exhibited the increased HA level (Table 1), and CBS is a calcium-dependent enzyme [15]. 3xTg-AD model mice exhibit the activated CBS activity, which induced the increased HA level. Figure 1 shows that memory impairment induced by HA is stronger in the presence of Aβ. However, Aβ itself does not induce memory impairment because transgenic experimental mice, who had higher levels of Aβ-40 and −42 display better memory performance than non-Tg mice. Recently, it has been reported that amyloid oligomers induce long-term potentiation suppression through the action of NMDA [17]; this finding supports our observations. Our observations indicate that anti-HA antibody can bring about normal recovery in AD. It can therefore be concluded that HA is a true etiological agent for AD. Our hypothesis that HA accelerated the pathogenesis of AD was confirmed by the immunohistochemical observations. Aβ accumulated in hippocampal neurons in the transgenic control group but not in the transgenic experimental group. The curative effect of anti-HA antibody was so strong that a substantive recovery in memory performance tasks was observed. HA induced memory impairment in our study and this impairment was greater in the presence of Aβ (Fig. 1), i.e., even if Aβ were to be decreased, HA would still be present in the affected brain tissue. Our findings on anti-HA antibody treatment in transgenic mice demonstrate that the toxicity of HA is decreased and neurodegeneration is inhibited following which hippocampus volume increased, suggesting that neurogenesis may occur (Table 4).

Now, we would like to clarify whether the HA vaccine (anti-HA antibody) induced neurogenesis. We are interested in the finding that the anti-pain effect induced the neurogenesis of hippocampus [10]. Results show the strong anti-pain effect of HA vaccine (Table 5), which suggests that HA vaccine (anti-HA antibody) induced the neurogenesis of hippocampus; this suggestion is consistent with our observation that the hippocampal volume of the experimental mice is larger than that of the control mice. This led to the recovery of memory performance to a normal state. It has been recently reported that NMDA antagonists such as MK-801 induce neurogenesis in the dentate gyrus of the hippocampus [16] and that HA is an agonist of the NMDA receptor [2]. Then anti-HA antibody and HA vaccine also may induce neurogenesis according to the observation of Qu K et al. [15]

In conclusion, (i) HA accelerates pathological changes in AD; and (ii) HA toxicity decreases with anti-HA antibody, which induces strong neurogenesis in the hippocampus, resulting in marked recovery of memory performance. Our hypothesis is also supported by the observation that anti-HA antibodies induced marked recovery in 7-month-old hemizygous mice However, recovery was observed on the second trial day and not the third trial day. This observation could be probably induced by the fact that the control hemizygous mice did not impair partially their memory ability at 7 months old and that control mice exhibited partial good memory performance on the third trial day. We should note the cure effect of anti-HA antibody or HA vaccine on memory impairment of 3xTg-AD mice. Homozygous 3xTg-AD mice exhibited complete memory impairment at 12 months old with normal food intake (Fig. 5). HA vaccine immunized homozygous male 3xTg-AD mice at 12 months and their memory performance exhibited complete recovery compared with normal 2-month-old mice.

This is the first study, to our knowledge, to demonstrate marked recovery from AD induced by treatment with anti-HA antibody or HA vaccine. Our findings prove the strong curative effect of anti-HA antibody treatment and HA vaccine and support the idea that HA is a true etiological agent and an accelerator in the pathogenesis of AD. However, one may think that vitamin B6-deficient burden 3xTg-AD mice to have HA pathogen artificially. We thus needed to investigate whether the effect of anti-HA antibody or HA vaccine could be observed in the normal feeding of 3xTg-AD mice. The result shows clearly the strong cure effect of anti-HA antibody and HA vaccine in the normal feeding of 3xTg-AD mice (12-month-old; Fig. 5).

But how does HA induce neurodegeneration? HA affects the two pathophysiological hallmarks of AD and may be involved in its etiology. Moreover, HA itself can induce neurodegeneration at a higher level with no amyloid [4], and the HA toxicity is induced by calcium influx [17] or by mitochondrial inhibition [5] or oxidative stress [4].
